# Percutaneous Radiofrequency Ablation Is an Effective Method for Local Control of Liver Metastases From Lung Cancer

**DOI:** 10.3389/fonc.2022.877273

**Published:** 2022-04-06

**Authors:** Zhong-Yi Zhang, An-Na Jiang, Wei Yang, Kun Yan, Wei Wu, Song Wang, Bin-Bin Jiang, Li-Qi Sun, Kun Zhao, Min-Hua Chen

**Affiliations:** Key Laboratory of Carcinogenesis and Translational Research (Ministry of Education/Beijing), Department of Ultrasound, Peking University Cancer Hospital & Institute, Beijing, China

**Keywords:** lung cancer, liver metastasis, radiofrequency ablation, prognosis, clinical value

## Abstract

**Objective:**

To investigate the clinical value of percutaneous radiofrequency ablation (RFA) for liver metastasis from lung cancer (LCLM).

**Materials and Methods:**

We retrospectively enrolled 58 patients who underwent RFA for LCLM between January 2014 and December 2019. Primary lung cancer histology included 38 adenocarcinomas, 15 squamous carcinomas, and 5 small cell carcinomas. For 83 metastatic lesions (mean tumor diameter 3.3 ± 1.1 cm, range 0.9–5.0 cm), 65 RFA sessions were performed. Before RFA, 17 and 41 patients presented no and stable extrahepatic metastasis, respectively, whereas 18 and 40 patients had synchronous and metachronous liver metastasis, respectively. Survival was analyzed using the Kaplan-Meier method. Cox proportional hazards model was used for multivariable analysis.

**Results:**

The technical success rate was 96.3% (80/83 lesions). Local tumor progression was observed in 8 (9.8%, 8/82) lesions of 57 (14.0%, 8/57) patients at 4–12 months after RFA. New liver metastases occurred in 27 (46.6%) patients. The overall survival (OS) rates at 1, 2, 3, and 5 years after RFA were 55.2%, 26.0%, 22.0%, and 14.4%, respectively. The median OS after RFA and after liver metastasis were 14.0 ± 1.6 and 20.0 ± 1.5 months, respectively. Based on the univariable analysis, tumor size (p=0.017), histological type (p=0.015), and timing of liver metastasis (p=0.046) were related to OS. In further multivariable analyses, squamous carcinoma (hazard ratio= 2.269, 95% confidence interval: 1.186-4.339, p=0.013) was an independent unfavorable prognostic factor for OS. Based on the univariable analysis, histological type (p=0.010) was identified as parameters significantly related to local tumor progression (LTP)-free survival. Further multivariable analyses revealed that squamous carcinoma (hazard ratio=2.394, 95% confidence interval: 1.260–4.550, p=0.008) was an independent unfavorable prognostic factor for LTP-free survival.

**Conclusion:**

RFA is a safe therapeutic option for LCLM with acceptable local tumor control, especially in patients with a tumor size ≤3 cm, adenocarcinoma/small cell carcinoma, and metachronous liver metastases.

## Introduction

Lung cancer is the leading cause of cancer incidence and mortality worldwide (1). In patients with lung cancer, the liver is one of the most common metastatic sites with a reported incidence of 37–51% in autopsies (2). Liver metastasis is an unfavorable prognostic factor, with a median overall survival (OS) of 3–12 months (3–6). Clinically, systemic therapy, including chemotherapy, target therapy, and immune therapy, is recommended for metastatic lung cancer. However, liver metastases are usually associated with an unfavorable response to systemic therapy. For those patients, removal of liver metastases provides a therapeutic option to improve the prognosis. It has been reported that liver resection can offer long-term survival in selected patients with lung cancer liver metastasis (LCLM) (7, 8).

Percutaneous radiofrequency ablation (RFA) has been widely used for hepatocellular carcinoma and colorectal liver metastasis and has become an alternative treatment option. RFA has been proven to provide good safety and local tumor control while preserving liver parenchyma. The retrospective study by Tseng et al. (9) reported that pulmonary adenocarcinoma patients with liver metastasis who received RFA had longer OS than those without RFA. Zhao et al. (10) retrospectively reported systemic therapy plus thermal ablation could prolong progression-free survival but not OS in liver metastases from non-small cell lung cancer (NSCLC). However, there are still not many studies on the effectiveness of RFA in patients with LCLM. Therefore, the present study summarizes a single-center experience of ultrasound-guided percutaneous RFA for LCLM.

## Materials and Methods

### Patients

We retrospectively analyzed data of 78 patients with LCLM who underwent ultrasound-guided percutaneous RFA in our center between January 2014 and December 2019. Before RFA treatment, each patient signed an informed consent form. The Institutional Review Board waived the protocol authorization requirement due to the retrospective nature of this study.

**Figure 1:**
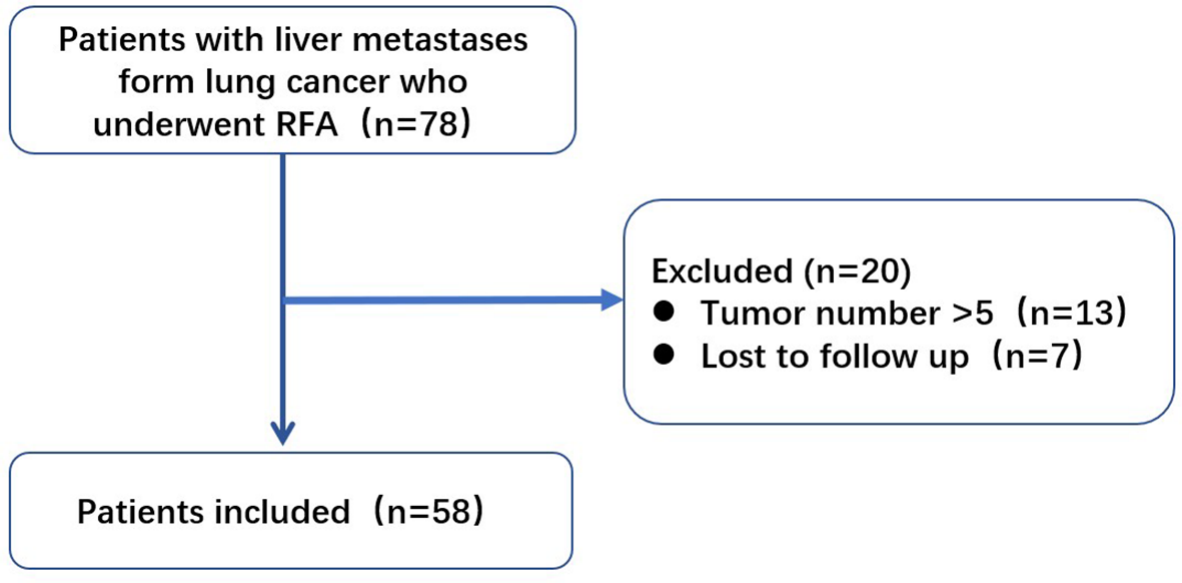
Flow diagram of patient selection.

Of the initially enrolled patients, 13 were excluded because of tumor numbers >5, and 7 were lost to follow-up. The remaining 58 patients were included in this study (**Figure 1**). All patients met the following criteria for RFA (1): liver metastasis visible on conventional ultrasonography or contrast-enhanced ultrasonography (2), tumor number ≤5, with maximum diameter ≤5 cm (3), a liver function classification of Child-Pugh A or B (4), no direct invasion of adjacent organs or tumor thrombi in the main or lobar portal system (5), absence of main bile duct invasion (6), platelet count >50000/μl and prothrombin activity >50% (7), extrahepatic metastases, if present, controlled or stable before RFA, and (8) patients were willing to receive local therapy for liver metastases. The decision to perform RFA was reached by consensus from an interdisciplinary panel consisting of surgeons, oncologists, radiologists, radiation therapists, and pathologists. In our center, RFA has been widely used for liver malignancies in the last 20 years, and RFA is recommended if liver metastasis is limited and extrahepatic metastasis is controlled. The doctors thought the patients might get benefit from RFA treatment because they considered liver metastasis might threaten patients’ life than other sites disease.

Based on the pathological findings of the pulmonary lesion, primary lung cancer was diagnosed as adenocarcinoma (n=38), squamous carcinoma (n=15), or small cell carcinoma (n=5). The liver metastases were evaluated based on typical imaging findings, serum tumor markers, and clinical history. Among the 58 analyzed patients, 9 received liver biopsies before RFA, and the pathological diagnosis confirmed liver metastases from lung cancer.

The 58 patients included 37 males and 21 females with an average age of 61.0 ± 10.0 (range: 33–78) years. A total of 83 metastases underwent RFA in 65 sessions. The mean tumor diameter of the metastases was 3.3 ± 1.1 (range 0.9–5.0) cm. In this study population, 47 patients had a single liver metastasis, whereas 11 patients had multiple liver metastases. Before RFA, there were 17 patients without extrahepatic metastasis and 41 patients with extrahepatic metastasis, including 25 patients with intrathoracic metastases, 11 patients with brain metastases, and 23 patients with bone metastases. Synchronous liver metastasis was defined as metastasis identified at the time of primary lung cancer diagnosis or within 6 months after the primary lung cancer had been diagnosed. Metachronous liver metastasis was defined as metastasis detected at least 6 months following the primary lung cancer diagnosis. Among the 58 analyzed patients, 18 patients had synchronous liver metastasis, and 40 patients had metachronous liver metastasis. Before RFA treatment, 25 patients underwent resection of the primary lung cancer, whereas 33 patients did not receive pulmonary surgery. Within the 3 months before RFA treatment, 13 patients did not receive any systemic therapy (chemotherapy and/or target therapy). Among the 45 patients undergoing systemic therapy, liver metastases showed a partial response in 12 patients, stable disease in 6 patients, and progressive disease in 27 patients according to the RECIST criteria (11).

### RFA Procedure

All RFA procedures were performed by two radiologists with more than 10 years of experience in ultrasound-guided interventional procedures. RFA systems used in this study was internally cooled multipolar (CelonLab POWER system, Teltow, Germany) electrodes. The CelonLab POWER system provides a maximum power output of 250W and can connect up to three 16-gauge electrodes with an exposed tip of 2-4cm. The size of coagulation depends on the exposed tip length, electrode number and distance between the electrodes.

Prosound α-10 (ALOKA, Tokyo, Japan), and Logic E9 (GE Healthcare, Chicago, USA) ultrasound systems were used for scanning and guidance.

The ablation procedure has been described in detail previously (12, 13). Contrast-enhanced computed tomography (CECT) and/or magnetic resonance imaging (MRI) were performed within 1 month prior to RFA and were used as a reference for RFA planning. Contrast-enhanced ultrasound was routinely performed by using Sonove (Bracco, SPA, Italy) during RFA. Patients were placed in the supine or oblique position depending on the tumor location. Sedative anesthesia was induced by using 2.5-5mg of midazolam (Roche, Basel, Switzerland) and 50-100μg of fentanyl (Fentaini, Renfu, Yichang, China). Local infiltration anesthesia was performed by 1% lidocaine (Liduokayin, Beijing, China) and then electrodes were inserted into the tumor under ultrasound guidance. For tumors >3 cm, overlapping ablations were used. The ablation zone aimed to cover a distance of at least 0.5 cm beyond the tumor. During the procedure, the vital signs of the patients were continuously monitored. After treatment, tract ablation was performed when withdrawing the electrode. After RFA, contrast-enhanced ultrasound (CEUS)was immediately performed to evaluate the ablation zone. If a residual tumor was detected, supplementary ablation was performed. The patients were admitted on the day of RFA and stay in hospital overnight after RFA. If any major complication was not observed, the patients would be discharged the next day.

### Assessment of Efficacy and Follow-Up

CECT or MRI results at 1 month after the treatment were used to determine the technical success rate. Technical success was defined as the ablation zone displaying no enhancement on CECT or MRI and completely covering the position of primary liver metastasis. By contrast, technical failure refers to any abnormal enhancement observed within or near the ablation target. Subsequently, CECT or MRI was performed every 3 months in the first year and every 4–6 months thereafter. Local tumor progression (LTP) was defined as the appearance of new metastasis along the ablation zone at least 1 month after RFA treatment. When the new metastasis appeared far away from the ablation zone, it was defined as new tumor development.

### Safety Assessment

Complications were recorded according to the Society of Interventional Radiology clinical practice guidelines (14) during the follow-up. Major complications were defined as events threatening the life of a patient, causing disability, requiring hospitalization, or prolonging hospitalization.

### Statistical Analyses

Continuous variables are presented as the mean ± standard deviation. Survival was analyzed using the Kaplan-Meier method. Eleven potential prognostic factors were analyzed, including age, tumor size, number, extrahepatic metastasis, intrathoracic metastasis, brain metastasis, bone metastasis, histological type, timing of liver metastasis, resection of lung cancer, and liver tumor response to systemic therapy before RFA. The log-rank test was used for univariable analyses. If p<0.1 in log-rank test, the factor was selected for further multivariable analyses. The Cox proportional hazards model was used for multivariable analyses. STATA 17 software was employed for all statistical analyses, and p<0.05 was considered statistically significant.

## Results

### Technical Efficacy, Local Tumor Progression, and New Tumor Development

The technical success rate was 96.4% (80/83 lesions). Residual tumors occurred in 3 lesions of 3 patients, including 3 tumors larger than 3 cm, 3 tumors close to the secondary portal vein or hepatic vein, 1 tumor close to the digestive tract and diaphragm. Repeat RFA was administered in 2 patients, achieving technical success. The remaining patient received chemotherapy due to the occurrence of multiple metastases.

The median follow-up time in this study was 13.5 months. During the follow-up, LTP was observed in 8 (9.8%, 8/82) lesions of 57 (14.0%, 8/57) patients at 4–12 months after RFA. Repeat RFA was administered in 1 patient achieving technical success, whereas 7 patients received systemic therapy. A total of 27 (46.6%) patients developed new liver metastases after a median of 7 (range: 2–64) months following RFA and received further repeat RFA (1 patient), transcatheter arterial chemoembolization (2 patients), and systemic therapy (24 patients).

### Survival Rate and Prognostic Analysis

At the last follow-up in March 2021, 11 patients were alive, whereas the remaining 47 patients had died. The OS rates at 1, 2, 3, and 5 years after RFA were 55.2%, 26.0%, 22.0%, and 14.4%, respectively. The median OS after RFA and after liver metastasis were 14.0 ± 1.6 and 20.0 ± 1.5 months, respectively. The LTP-free survival rates at 1, 2, 3, and 5 years after RFA were 50.0%, 24.4%, 20.3%, and 13.1%, respectively. The median LTP-free survival after RFA was 12.0 ± 1.9 months.

**Figure 2:**
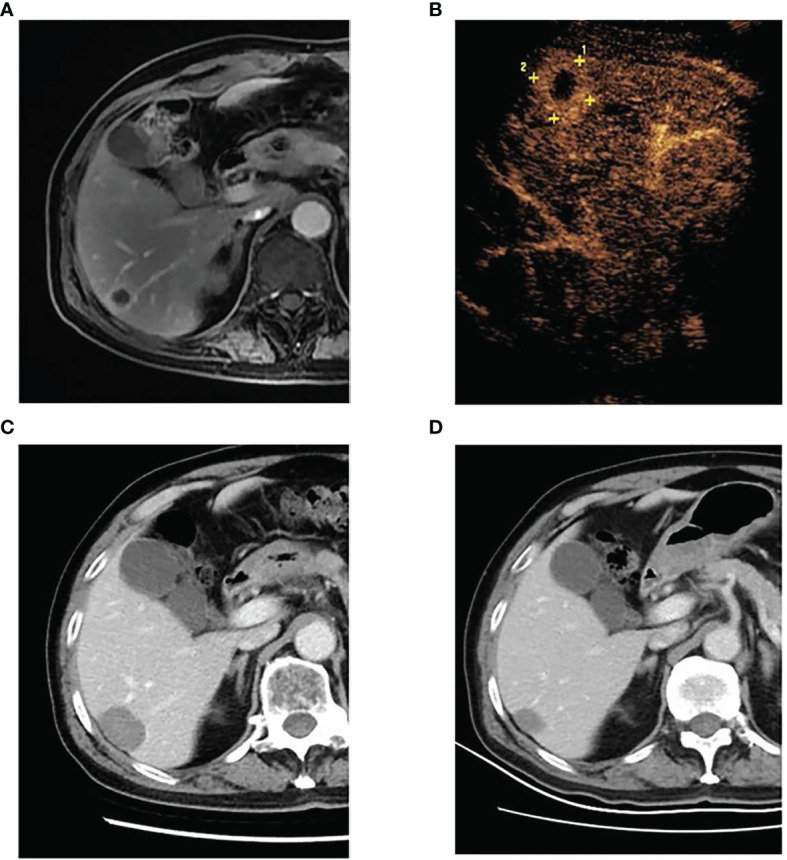
A 68-year-old man was diagnosed with left lung adenocarcinoma and received radiotherapy and chemotherapy for the lung lesion. After 27 months, metachronous liver metastasis was detected by magnetic resonance imaging. The patient underwent percutaneous radiofrequency ablation for liver metastasis. Until the last follow-up, the patient was alive, surviving 30 months without recurrence. **(A)** The liver metastasis in segment VI, as detected by magnetic resonance imaging. **(B)** Contrast-enhanced ultrasound showing a 2.1-cm liver tumor in segment VI. **(C)** Contrast-enhanced computed tomography at 1 month after radiofrequency ablation demonstrating the successful ablation of the tumor. **(D)** Contrast-enhanced computed tomography at 30 months after radiofrequency ablation showing that the ablation zone shrunk without local recurrence or new liver metastases.

**Figure 3:**
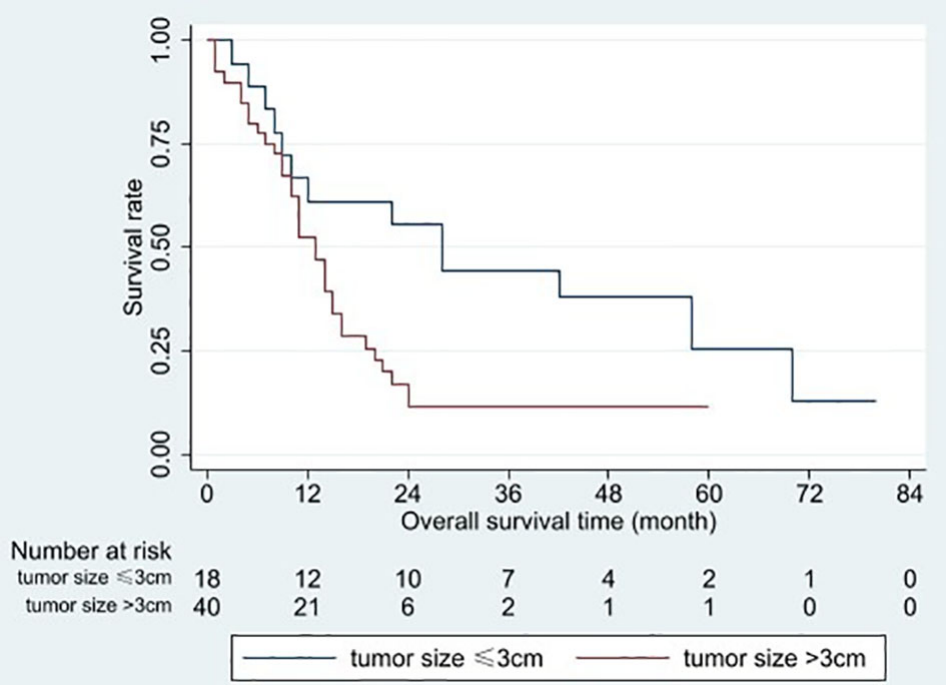
Overall survival curves for different tumor sizes. Patients with a tumor ≤3 cm had better overall survival than those with a tumor >3 cm (p = 0.017).

**Figure 4:**
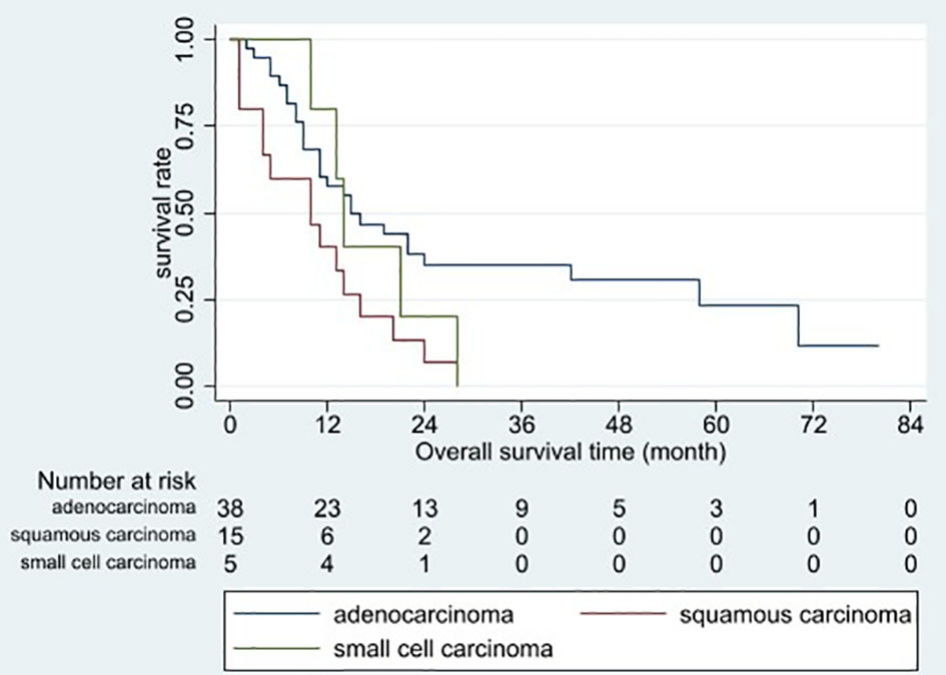
Overall survival curves for different histopathological tumor types. Patients with squamous carcinoma had the poorest overall prognosis (p = 0.015).

**Figure 5:**
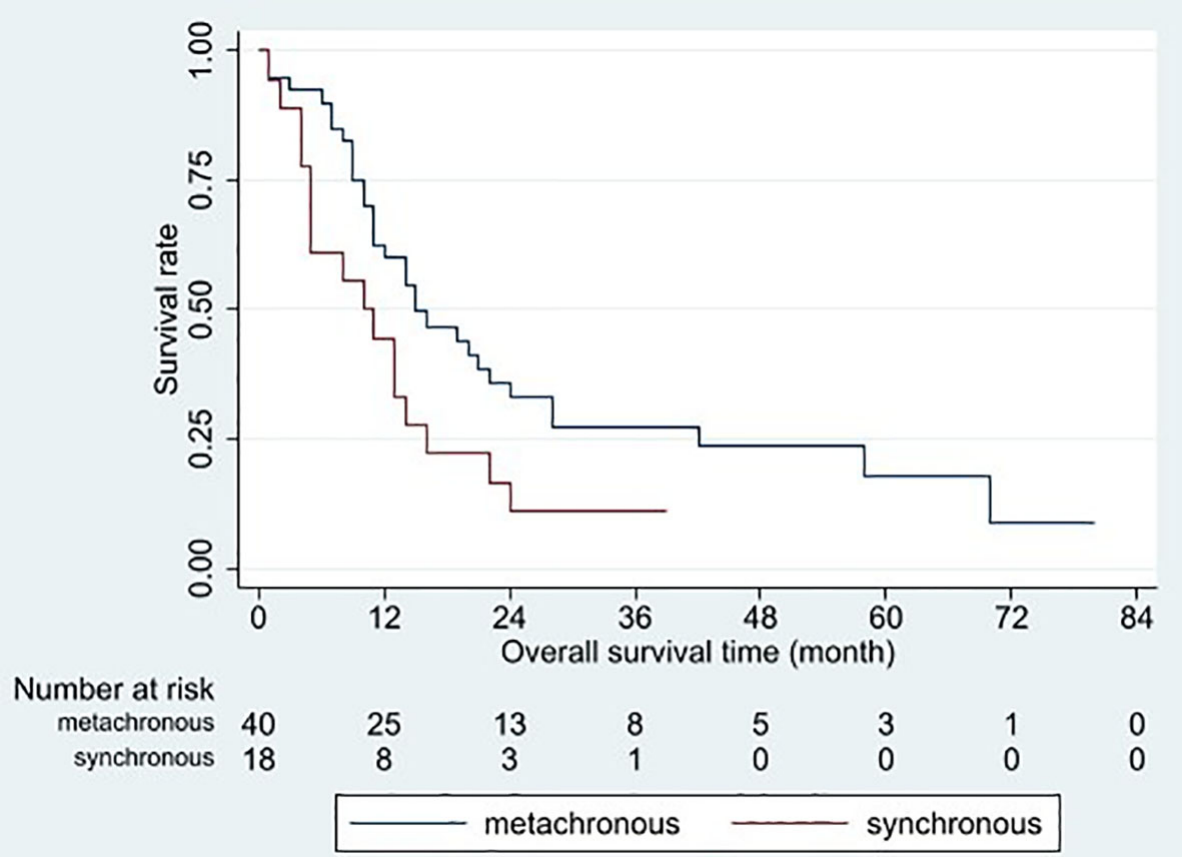
Overall survival curves for different timings of liver metastasis occurrences. Patients with metachronous metastasis had better overall survival than those with synchronous metastasis (p = 0.046).

Based on the univariable analysis, tumor size (p=0.017), histological type (p=0.015), and timing of liver metastasis (p=0.046) were identified as parameters significantly related to OS (**Table 1** and **Figures 2**–**5**). Further multivariable analyses revealed that squamous carcinoma (hazard ratio [HR]= 2.269, 95% confidence interval [CI]: 1.186-4.339, p=0.013) was an independent unfavorable prognostic factor for OS (**Table 2**).

**Table 1:** Univariable analysis of liver metastasis of lung cancer on overall survival.

Factor	Survival						
	No	1 year (%)	2 year (%)	3 year (%)	5 year (%)	median (month)	P value
age							0.748
≤60 years	27	55.6	22.2	17.8	8.9	14	
>60 years	31	61.3	29.7	26.0	17.3	15	
tumor size							0.017
≤3cm	18	61.1	55.6	44.4	25.4	28	
>3cm	40	52.5	17.1	11.4	11.4	13	
tumor number							0.510
1	47	53.2	24.7	19.8	16.5	14	
≥2	11	63.6	31.8	31.8	15.9	16	
extrahepatic metastasis							0.597
no	17	58.8	29.4	22.1	22.1	16	
yes	41	53.7	24.6	21.9	13.7	13	
intrathoracic metastasis							0.169
no	33	60.6	34.0	26.5	21.2	16	
yes	25	48.0	16.0	16.0	10.7	11	
brain metastasis							0.388
no	47	55.3	25.5	20.9	11.9	14	
yes	11	54.5	29.1	29.1	29.1	14	
bone metastasis							0.464
no	35	57.1	21.1	17.5	0	14	
yes	23	52.2	33.5	28.7	23.0	14	
histological type							0.015
adenocarcinoma	38	57.9	35.0	35.0	23.0	15	–
squamous carcinoma	15	40.0	6.7	0	0	10	0.005*
small cell carcinoma	5	80.0	20.0	0	0	14	0.386*
timing of liver metastasis							0.046
Metachronous	40	60.0	33.0	27.0	17.7	15	
Synchronous	18	44.4	11.1	11.1	11.1	10	
resection of primary lung cancer							0.953
yes	25	64.0	28.0	18.7	12.4	16	
no	33	48.5	24.9	24.9	18.7	11	
liver tumor response of systemic therapy							0.850
without systemic therapy	13	53.8	23.1	23.1	23.1	14	–
PR	12	66.7	33.3	16.7	0	14	0.979#
SD/PD	33	51.5	24.3	24.3	24.3	13	0.942#

*compared with subgroup of ‘adenocarcinoma’.

#compared with subgroup of ‘without systemic therapy’.

**Table 2:** Multivariable analysis of liver metastasis of lung cancer on overall survival.

factor	Wald value	HR	95% CI	P value
>3cm	2.263	1.759	0.843-3.670	0.132
squamous carcinoma	6.130	2.269	1.186-4.339	0.013
synchronous liver metastases	2.439	1.682	0.876-3.231	0.118

Based on the univariable analysis, histological type (p=0.010) was identified as parameters significantly related to LTP-free survival. Further multivariable analyses revealed that squamous carcinoma (hazard ratio [HR]=2.394, 95% confidence interval [CI]: 1.260–4.550, p=0.008) was an independent unfavorable prognostic factor for LTP-free survival.

### Complications

No patient experienced death related to RFA. The occurrence of major complications was 1.5% (1/65 RFA sessions). One patient experienced intestinal obstruction and recovered following conservative treatment.

## Discussion

Distant metastases of lung cancer represent a frequent clinical problem. Clinically, systemic therapy is recommended for metastatic lung cancer but some patients do not respond well. In the present study, only 20.7% (12/58) of the patients had a partial response to systemic therapy before RFA. Because several cytotoxic agents used in the treatment of lung cancer are activated or metabolized by the liver, some patients with liver metastases are also unable to continue systemic therapy due to liver dysfunction. These patients may benefit from liver metastasis removal.

Zhao et al. (10) reported systemic therapy plus thermal ablation (n=21) could prolong PFS compared with systemic therapy alone (n=40; 11.0 vs 5.2 months, respectively; p=0.001) in NSCLC patients with liver metastases. Tseng et al. (9) retrospectively analyzed data of pulmonary adenocarcinoma patients with liver metastasis. Patients who received RFA (n=6) had longer OS after liver metastasis than those without RFA (n=42; 23.1 vs 7.9 months, respectively; p=0.035). Similarly with the study of Tseng et al. the median OS after liver metastasis in present study was 20.0 months, which was longer than that determined in previous studies for patients without local therapy (median OS: 3–12 months) (3–6). This indicates that RFA might be effective to prolong OS in patients with LCLM.

The LTP after RFA in patients with liver metastasis varies between 8.8% and 55.0% (15, 16). In the present study, it was 9.8% which is at the lower end of the range in previous studies. This can be explained by the fact that the ablative margin was at least more than 5 mm in the present study. Shady et al. (17) compared the effects of different ablative margins on LTP-free survival rates after RFA of colorectal liver metastases. These rates for tumors with a margin >5–10 mm were significantly lower than those with a margin ≤5 mm (71% vs 14.8%, respectively; p<0.001). No LTP was observed for tumors ablated with a margin >10 mm. To achieve the best local tumor control, the ablative margin should exceed 5 mm or even >10 mm, if possible.

Riihimäki et al. (6) reported that squamous carcinoma conferred a slightly worse prognosis than adenocarcinoma (HR: 1.1, 95% CI: 1.0–1.21, p>0.05). In the present study, LCLM with squamous carcinoma had a poorer prognosis than LCLM with adenocarcinoma or small cell carcinoma (median OS: 10 vs 15 vs 14 months, p=0.015). Differences in biological behavior and response to systemic therapy may explain these discrepancies among histological tumor types. Regarding the small sample size of the present study, further studies with larger sample sizes may confirm the study results.

The present study identified a tumor size >3 cm as an unfavorable factor for LCLM. OS after RFA was significantly shorter in patients with a tumor size >3 cm than in those with a tumor size ≤3 cm (13 vs 28 months, respectively; p=0.017). The results of previous studies were similar for liver metastases from colorectal cancer (18) and breast cancer (12). In addition to the increased tumor burden in patients with a tumor size >3 cm, overlapping ablations were usually used. This makes it more difficult to ensure adequate ablative margins in three dimensions, which might contribute to reduced OS.

Synchronous metastasis was another unfavorable prognostic factor. The meta-analysis by Ashworth et al. yielded similar results (19). By contrast, there was no difference in median OS between synchronous and metachronous oligometastases groups (HR: 1.26, 95% CI: 0.71–2.24, p=0.43) in the study of Fleckenstein et al. (20). However, most patients in these studies had brain and lung metastases, whereas liver metastases only accounted for less than 5%. It is still a lack of studies focus on the timing of the appearance of liver metastases.

This study has several limitations as follows. First, it was a single-center retrospective study with limited sample size, although, to the best of our knowledge, the present study has the largest study population focusing on RFA for LCLM. Second, the median OS of patients after the detection of liver metastasis was 20 months in this study, which was significantly prolonged compared to 3–12 months in patients who did not receive local therapy in previous studies (3–6). However, due to the lack of patients with liver metastasis who did not receive RFA, it is difficult to evaluate whether RFA really improves OS. Further prospective trails are needed to evaluate the effectiveness of RFA for LCLM.

In conclusion, RFA is a safe therapeutic option for LCLM with acceptable local tumor control, especially in patients with a tumor size ≤3 cm, adenocarcinoma/small cell carcinoma, and metachronous liver metastases.

## Data Availability Statement

The raw data supporting the conclusions of this article will be made available by the authors, without undue reservation.

## Ethics Statement

The studies involving human participants were reviewed and approved by Peking University Cancer Hospital & Institute. The ethics committee waived the requirement of written informed consent for participation.

## Author Contributions

Z-YZ, WY, SW, WW, and KY performed RFA. A-NJ, B-BJ, L-QS, and KZ helped to collect the data and follow-up. A-NJ wrote the draft version of this paper, analyzed the data and revised it with Z-YZ. WY helped to revise the paper and gave some important opinions. M-HC gave some important opinions about this research. All authors have read and approved the manuscript.

## Funding

This study was supported by National Natural Science Foundation of China (81971718), Natural Science Foundation of Beijing Municipality (7222020) and Capital’s Funds for Health Improvement and Research (2020–2–2152).

## Conflict of Interest

The authors declare that the research was conducted in the absence of any commercial or financial relationships that could be construed as a potential conflict of interest.

## Publisher’s Note

All claims expressed in this article are solely those of the authors and do not necessarily represent those of their affiliated organizations, or those of the publisher, the editors and the reviewers. Any product that may be evaluated in this article, or claim that may be made by its manufacturer, is not guaranteed or endorsed by the publisher.

## References

[1] Bray F Ferlay J Soerjomataram I Siegel RL Torre LA Jemal A. Global Cancer Statistics 2018: GLOBOCAN Estimates of Incidence and Mortality Worldwide for 36 Cancers in 185 Countries. CA Cancer J Clin (2018) 68:394–424. doi: 10.3322/caac.21492 30207593

[2] Tas F Aydiner A Topuz E Camlica H Saip P Eralp Y. Factors Influencing the Distribution of Metastases and Survival in Extensive Disease Small Cell Lung Cancer. Acta Oncol (1999) 38:1011–5. doi: 10.1080/028418699432275 10665754

[3] Tamura T Kurishima K Nakazawa K Kagohashi K Ishikawa H Satoh H. Specific Organ Metastases and Survival in Metastatic Non−Small−Cell Lung Cancer. Mol Clin Oncol (2015) 3:217–21. doi: 10.3892/mco.2014.410 PMC4251107 25469298

[4] Riihimäki M Hemminki A Fallah M Thomsen H Sundquist K Sundquist J. Metastatic Sites and Survival in Lung Cancer. Lung Cancer (2014) 86:78–84. doi: 10.1016/j.lungcan.2014.07.020 25130083

[5] Brueckl WM Ficker JH Zeitler G. Clinically Relevant Prognostic and Predictive Markers for Immune-Checkpoint-Inhibitor (ICI) Therapy in Non-Small Cell Lung Cancer (NSCLC). BMC Cancer (2020) 20:1185. doi: 10.1186/s12885-020-07690-8 33272262 PMC7713034

[6] Liao Y Fan XM Wang X. Effects of Different Metastasis Patterns, Surgery and Other Factors on the Prognosis of Patients With Stage IV Non-Small Cell Lung Cancer. Oncol Lett (2019) 18:581–92. doi: 10.3892/ol.2019.10373 PMC6546983 31289530

[7] Nagashima A Abe Y Yamada S Nakagawa M Yoshimatsu T. Long-Term Survival After Surgical Resection of Liver Metastasis From Lung Cancer. Jpn J Thorac Cardiovasc Surg (2004) 52:311–3. doi: 10.1007/s11748-004-0050-y 15242087

[8] Ileana E Greillier L Moutardier V Barlesi F. Surgical Resection of Liver Non-Small Cell Lung Cancer Metastasis: A Dual Weapon? Lung Cancer (2010) 70:221–2. doi: 10.1016/j.lungcan.2010.08.010 20828859

[9] Tseng SE Chiou YY Lee YC Perng RP Jacqueline WP Chen YM. Number of Liver Metastatic Nodules Affects Treatment Options for Pulmonary Adenocarcinoma Patients With Liver Metastases. Lung Cancer (2014) 86:225–30. doi: 10.1016/j.lungcan.2014.09.002 25240517

[10] Zhao YQ Zhang XW Zhao H Gong T Li JG Tsauo JW. Systemic Therapy Plus Thermal Ablation Versus Systemic Therapy Alone for Oligometastatic Liver Metastases From non-Small Cell Lung Cancer. Cardiovasc Intervent Radiol (2020) 43:1285–93. doi: 10.1007/s00270-020-02456-y 32236671

[11] Eisenhauer EA Therasse P Bogaerts J Schwartz LH Sargent D Ford R. New Response Evaluation Criteria in Solid Tumours: Revised RECIST Guideline (Version 1.1). Eur J Cancer (2009) 45:228–47. doi: 10.1016/j.ejca.2008.10.026 19097774

[12] Bai XM Yang W Zhang ZY Jiang AN Wu W Lee JC. Long-Term Outcomes and Prognostic Analysis of Percutaneous Radiofrequency Ablation in Liver Metastasis From Breast Cancer. Int J Hyperthermia (2019) 35:183–93. doi: 10.1080/02656736.2018.1488279 30200791

[13] Jiang BB Yan K Zhang ZY Yang W Wu W Yin SS. The Value of KRAS Gene Status in Predicting Local Tumor Progression of Colorectal Liver Metastases Following Radiofrequency Ablation. Int J Hyperthermia (2019) 36:211–9. doi: 10.1080/02656736.2018.1556818 30663903

[14] Sacks D McClenny TE Cardella JF Lewis CA. Society of Interventional Radiology Clinical Practice Guidelines. J Vasc Interv Radiol (2003) 14:S199–202. doi: 10.1097/01.RVI.0000094584.83406.3e 14514818

[15] Minami Y Kudo M. Radiofrequency Ablation of Liver Metastases From Colorectal Cancer: A Literature Review. Gut Liver (2013) 7:1–6. doi: 10.5009/gnl.2013.7.1.1 23422905 PMC3572308

[16] Bale R Putzer D Schullian P. Local Treatment of Breast Cancer Liver Metastasis. Cancers (Basel) (2019) 11:1341. doi: 10.3390/cancers11091341 PMC6770644 31514362

[17] Shady W Petre EN Do KG Gonen M Yarmohammadi H Brown KB. Percutaneous Microwave Versus Radiofrequency Ablation of Colon Cancer Liver Metastases: Ablation With Clear Margins (A0) Provides the Best Local Tumor Control. J Vasc Interv Radiol (2018) 29:268–75. doi: 10.1016/j.jvir.2017.08.021 PMC5803367 29203394

[18] Gillams AR Lees WR. Five-Year Survival in 309 Patients With Colorectal Liver Metastases Treated With Radiofrequency Ablation. Eur Radiol (2009) 19:1206–13. doi: 10.1007/s00330-008-1258-5 19137310

[19] Ashworth AB Senan S Palma DA Riquet M Ahn YC Ricardi U. An Individual Patient Data Metaanalysis of Outcomes and Prognostic Factors After Treatment of Oligometastatic Non-Small-Cell Lung Cancer. Clin Lung Cancer (2014) 15:346–55. doi: 10.1016/j.cllc.2014.04.003 24894943

[20] Fleckenstein J Petroff A Schäfers HJ Wehler T Schöpe J Rübe C. Long-Term Outcomes in Radically Treated Synchronous vs Metachronous Oligometastatic Non-Small-Cell Lung Cancer. BMC Cancer (2016) 16:348. doi: 10.1186/s12885-016-2379-x 27255302 PMC4890277

